# Hypersensitivity to mesalazine in a patient with inflammatory bowel disease: a case report

**DOI:** 10.4076/1757-1626-2-6715

**Published:** 2009-08-25

**Authors:** Kevin C Cronin, Gabrielle C Colleran, Andrew Smyth, Diarmuid D Houlihan, Thomas A O’Gorman

**Affiliations:** 1 Department of Surgery, Galway University HospitalNewcastle Road, GalwayIreland; 2 Department of Medicine, Galway University HospitalNewcastle Road, GalwayIreland

## Abstract

A 32-year-old male presented with diarrhoea, mucus and bleeding per rectum. On the basis of sigmoidoscopy, rectal mesalazine was commenced uneventfully, and subsequently changed to oral mesalazine due to failure to improve.

He re-presented 4 days later with frequent rigors, lethargy, palpitations and was generally unwell. His condition settled with conservative management and IV antibiotics.

Oral mesalazine was withheld during the first 24 hours but was recommenced on day 2. After 2 doses he developed rigors, pyrexia, tachycardia and vomiting.

Oral mesalazine was discontinued thereafter and his condition progressively improved. Mesalazine has not been re-introduced.

## Case presentation

A 32-year-old male Caucasian healthcare worker presented with a 6/52 history of diarrhoea and mucus, and a 2/12 history or bleeding per rectum. He weighed 72.5 kg and was 1.78 m tall. Flexible sigmoidoscopy to the mid-sigmoid colon demonstrated granular mucosa with loss of the vascular pattern and bleeding into the mucosa. Rectal biopsies were taken. He was commenced on 4 g of Mesalazine enema per rectum nightly. He was reviewed two weeks later but remained symptomatic and oral mesalazine 1.2 g three times daily was initiated at this time.

He re-presented 4 days later with a significant clinical deterioration. He was febrile with frequent rigors, lethargic, complaining of palpitations and sinusitis. He was passing 8-10 stools per day with a significant amount of blood and mucus per rectum. He was on no other medications aside from the oral mesalazine.

His past medical history consisted of reflux as a child. He had no known food or drug allergies. His family history was significant for a maternal aunt with known colitis, a maternal uncle with ulcerative colitis and a mother with coeliac disease. He was a lifelong non-smoker and drank on average 15 units of alcohol per week.

On examination his temperature was 38.4°C, he was tachycardic, rigoring frequently and looked generally unwell.

He had routine blood tests and blood cultures carried out. He was subsequently admitted. He was commenced on IV co-amoxiclav and IV metronidazole. Stool cultures were sent for culture and sensitivity including Yersinia and Campylobacter. Both stool and blood cultures were reported as negative.

Overnight his temperature settled on the antibiotics. The frequency of bowel motions remained unchanged and his stools remained blood stained. His C reactive protein and his erythrocyte sedimentation rate were elevated. Oral mesalazine was not administered during the first 24 hours in hospital but was recommenced on the second day as an in-patient.

After 2 doses of oral mesalazine he developed rigors, high temperature, tachycardia and two episodes of vomiting. At the time sepsis was the major consideration but the possibility of a drug reaction was considered. The frequency of bowel motions increased to 15 per day. A second set of blood cultures was performed. He was commenced on piperacillin/tazobactam 4.5 g IV tds.

A Computed Tomography scan of abdomen with IV and oral contrast was carried out. This showed a thickened colonic wall, most marked in the rectum and distal sigmoid and also in the caecum. Some linear atelectasis was noted at the left lung base. These appearances were in keeping with the clinical diagnosis of a colitis with the distribution suggestive of a pancolitis most probably ulcerative colitis. IV hydrocortisone was commenced.

Oral mesalazine was discontinued after the second dose and he felt better post withdrawal of mesalazine. Both sets of blood cultures were provisionally reported as negative at this time. His clinical condition began to improve.

IV hydrocortisone was commenced on the fourth day of hospitalisation. 2 days later he was switched to oral prednisolone. He was discharged 3 days later on oral prednisolone.

As the oral steroids tapered he was started on azathioprine with stepwise augmentation of dose. His colitis reactivated and infliximab was introduced. He responded to infliximab and three months later infliximab was discontinued. He is currently well on azathioprine 150 mg orally once daily and iron once daily in oral liquid form. Mesalazine has not been re-introduced.

## Discussion

The rigors and general deterioration developed after the commencement of oral mesalazine. These ceased after mesalazine was withdrawn and recurred within hours of re-initiation of mesalazine. While it is possible that the pyrexia and rigors were related to an underlying sepsis there was no evidence of an abscess on the CT scan. Blood and stool cultures were negative. To confirm that the mesalazine was the cause of the fever it would be necessary to readminister a dose of the mesalazine. Neither the patient nor the physician felt that this was justified.

The 5-Aminosalicylates are important agents in the therapy of ulcerative colitis [1]. There is no advantage of mesalazine over sulfasalazine for serious adverse reactions [2]. Salicylate intolerance occurs in 2-7% of patients [3]. Gonzalo et al. report a case similar to ours where a patient who developed a fever response to mesalazine was successfully treated with a desensitizing programme to mesalazine with escalating doses to therapeutic level [4].

The natural history of ulcerative colitis is that of a chronic, lifelong condition with acute flare-ups and recovery. Intravenous corticosteroids are still the agent of choice in the treatment of an acute exacerbation of ulcerative colitis and the 5-aminosalicylates are normally the mainstay of treatment in the maintenance of remission in ulcerative colitis [5]. Azathioprine fulfils this role in this case due to the adverse reaction experienced by our patient to 5-ASA’s.

Other options for treatment in the acute phase include cyclosporine-A (CsA) although toxicity and the need for monitoring limit its use [6]. Recent advances in the understanding of the pathophysiology of inflammatory bowel disease have led to the development of a number of biological agents, of which infliximab, a monoclonal antibody to tumour necrosis factor alpha (TNF-α) was the first to be clinically available. Infliximab has a role both as an agent to maintain remission and as a rescue therapy in steroid-refractory disease [7]. For patients who remain refractory to all of these agents panproctocolectomy with or without reconstructive surgery will effect a cure but is not without its own complications [8].

The link between colitis and colorectal neoplasia is well established. In the long term our patient will require regular surveillance colonoscopy. There is a paucity of research on the long-term implications or cancer risk in patients in whom long-term 5-ASA therapy is not possible. The use of anti-inflammatory medications have been demonstrated to have a proven benefit to inhibiting the development of neoplastic lesions in animal models however this requires further work in-vivo [9]. Screening guidelines in UC are well established with a recommendation that colonoscopic screening should commence at 10 years from diagnosis [10].

## Conclusion

While allergic responses to Mesalazine are rare it is important to be aware that it is a potential cause of unexplained fever in patients with inflammatory bowel disease with no focus of infection.

## Abbreviation

CT: computed tomography
